# Reference values for feeding parameters of isopods (*Porcellio scaber*, Isopoda, Crustacea)

**DOI:** 10.3897/zookeys.457.6805

**Published:** 2014-11-25

**Authors:** Damjana Drobne, Samo Drobne

**Affiliations:** 1 University of Ljubljana, Biotechnical Faculty, Department of Biology, Vecna pot 111, SI-1000 Ljubljana, Slovenia; 2 University of Ljubljana, Faculty for Civil and Geodetic Engineering, Jamova 2, SI-1000 Ljubljana, Slovenia

**Keywords:** Feeding behaviour, toxicity testing, standard tests

## Abstract

The advantage of using terrestrial isopods in toxicity studies is that a battery of parameters can be tested at different levels of biological complexity. Feeding parameters for example link organism level response to potential ecological consequences but a problem with using feeding parameters in toxicity tests with terrestrial isopods is their high variability. The aim of our study was to set benchmark values for feeding parameters for isopod *Porcellio scaber* (Isopoda, Crustacea) in laboratory-controlled experiments. In the work presented here, the daily feeding rate of the central 50% of the control population of *Porcellio scaber* and a correlation between feeding rate and isopod weight were set. Values outside these ranges need additional evaluation to increase the relevance of test outcomes. We suggest using benchmark values for feeding parameters as well as the coefficient of variation (a) to identify animals with altered feeding parameters with respect to controls, and (b) to assess the data quality in each experiment.

## Introduction

Numerous studies have analysed feeding behaviour and food preferences of terrestrial isopods, which have an important ecological role as decomposers of leaf litter and are one of the model organisms used in laboratory toxicity testing and terrestrial ecotoxicology (Drobne and Hopkin 1994, Drobne 1997, Loureiro et al. 2006, Zidar et al. 2012).

Terrestrial isopods are saprophagous animals and have no single food source that meets all their nutritional needs. Only a mixture of several food items is an optimal nourishment (Zimmer 2002). In terrestrial environments, there is a significant imbalance of nutrients and in addition, many potential foods contain unfavourable concentrations of deterrents or other toxic compounds. Consequently, terrestrial isopods have acquired different feeding strategies, which allow them to respond to spatial and temporal variations in the quality and quantity of food (for review: Zimmer 2002).

Adaptations in feeding behaviour have allowed terrestrial isopods to reduce food consumption or be selective among different food items (Zidar et al. 2012). Their compensatory ability when consuming “low-quality” food to lengthen the food retention time in the gut is important (Wieser 1984). Such feeding behaviour responses to different foods are used in toxicity tests as organism level responses, which can be used to assess the effects of chemicals added to food (Drobne and Hopkin 1994, Drobne 1997, Loureiro et al. 2006). In toxicity studies, rates of feeding and faecal production as well as food assimilation efficiency are calculated (Drobne and Hopkin 1994, Loureiro et al. 2006, Novak et al. 2013). Reduction in feeding over that of control animals at a certain concentration of chemicals is taken as a measure of the effect at a given exposure dose (Drobne and Hopkin 1994). A significant benefit of using feeding parameters as toxicity endpoints is their potential to anticipate population and ecosystem consequences (De Coen and Janssen 2003, Lardies et al. 2004).

Since food consumption is indicative of the overall condition of an organism (De Coen and Janssen 2003), it is used as an indirect measure of fitness of the organism. It has been proven that in herbivorous arthropods, life history traits often depend upon diet quality. Lardies et al. (2004) provided evidence that in the terrestrial isopod *Porcellio laevis* the quality of dietary components has an effect on growth rate, size and number of offspring and incubation period, trade-offs between reproductive investment, growth, and survival, as well as the growth rate of offspring. Rushton and Hassall (1983) additionally reported that food quality affects population dynamics in isopods, presenting evidence that the growth rate of juvenile *Armadilldium vulgare* increased exponentially with the increasing proportion of high-quality food in the diet.

An intrinsic characteristic of feeding parameters is their relatively high variation. This fact is interpreted as “noise” when a desired signal is measured. As a result, the power of isopod toxicity tests with feeding rate as a measured response is reduced, and quantification of the response (signal) to toxicants is of limited power. In toxicity tests an adverse effect is proven when the signal-to-noise ratio equals or exceeds a certain value and when a clear dose-effect response is observed (Van der Lelie et al. 1997).

The aim of our study was to set the limits for feeding parameters of the terrestrial isopod *Porcellio scaber* (Isopoda, Crustacea) in laboratory-controlled experiments, which could serve as a benchmark for feeding rate of control, non-stressed animals. We analysed feeding parameters of about 600 animals in 60 experiments. In the analysed specimens, several sources of biological variability were retained to mimic to some degree the characteristics of isopod populations in nature. The sources of variability included: sex, moulting, different periods of laboratory acclimation, possible intracellular bacterial infection, and food quality. We discuss the interval for feeding rate, which could be taken as a characteristic for control animals – *i.e.* interquartile range – and suggest it as a benchmark for feeding rate of *Porcellio scaber* in a laboratory single-species test.

## Methods

### Model organisms

Terrestrial isopods (*Porcellio scaber*, Isopoda, Crustacea) were collected between June 2006 and June 2013 at different locations in Slovenia, which were all considered to be unpolluted. Prior to experiments, the animals were kept in a terrarium filled with a layer of moistened soil and a thick layer of partly decomposed hazelnut tree leaves (*Corylus avellana*), at a temperature of 20 ± 2 °C and a 16:8-h light:dark photoperiod. They were acclimated to laboratory conditions for at least 14 days before the start of the experiment. Adult animals of both sexes, weighing more than 25 mg, were used in the experiments.

### Experimental design (Drobne and Hopkin 1994, Novak et al. 2012)

Hazelnut leaves were dried at room temperature, cut into pieces weighed and placed in a Petri dish. One animal was placed in each dish together with two to four pieces of leaves, which were the isopods only food source. Petri dishes were kept in a large glass container under controlled conditions in terms of humidity (≥80%), temperature (21 ± 1 °C) and light regime (16:8-h light:dark photoperiod). After 14 days, the leaf remnants were removed, dried and weighed.

### Feeding parameter

Feeding rate per day (*FR*) was calculated as the amount of food consumed divided by the isopod fresh weight at the beginning of exposure (*IW*) per day.

### Data analysis

The data presented here were obtained from 60 different experiments conducted with 594 animals between 2006 and 2013. These specimens served as control animals in different feeding experiments. All experiments were conducted following the same exposure protocol, and standard operational procedure (Drobne and Hopkin 1994, Novak et al. 2013). The observed variables of the control animals, i.e. the weight of the animal at the beginning of experiment and the feeding rate per day, were analysed and tested using parametric and non-parametric statistical methods. The distribution of each variable was determined using the Kolmogorov-Smirnov test. The number of classes (*k*) for both variables was defined by Sturges rule for skewed data:


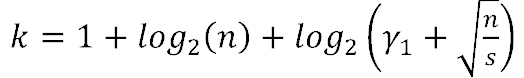
,

where *n* is the number of observations, *γ*_1_ is the coefficient of skewness, and *s* is standard deviation of observed data. Correlation between observed variables was tested by a t-test. The interquartile range of feeding rate per day was chosen arbitrarily as a reference interval for the control animals. Interquartile range defines the central 50% of sample. The interquartile range of observed variables of control animals was estimated by confidence intervals for the first (*Q*_1_) and the third quartile (*Q*_3_) using:


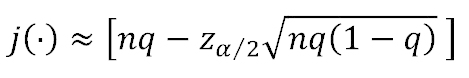



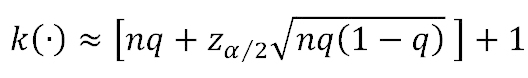
,

where *q* is the quantile rank of *Q*_1_ (*q*=0.25) and *Q*_3_ (*q*=0.75) respectively, *j* (*Q*_1_) and *j* (*Q*_3_) were ranks of lower limits of the confidence interval of *Q*_1_ respectively *Q*_3_, *k* (*Q*_1_) and *k* (*Q*_3_) were ranks of upper limits of confidence interval of *Q*_1_ respectively *Q*_3_. Confidence intervals for quartiles were estimated for risk level at α < 0.05, α < 0.01 and α < 0.001. Analysis and graphical presentations were performed in SPSS 21 and Excel 2013.

## Results

**Figure 1.**
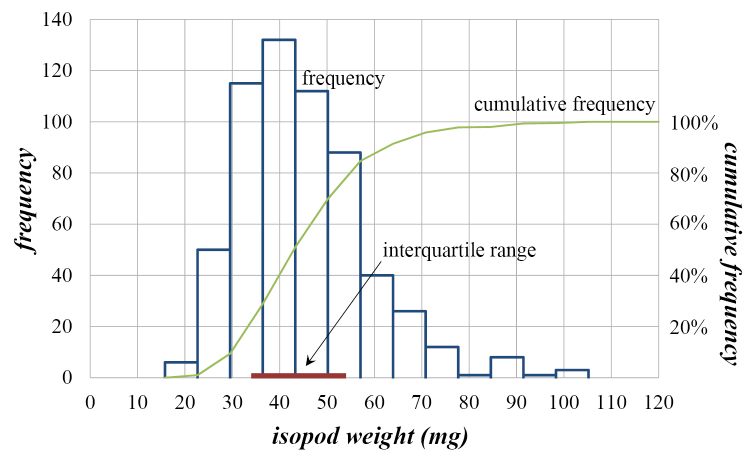
Frequency distributions and interquartile range of *Porcellio scaber* weight at the beginning of the experiment ([34< *IW*<54] mg).

**Figure 2.**
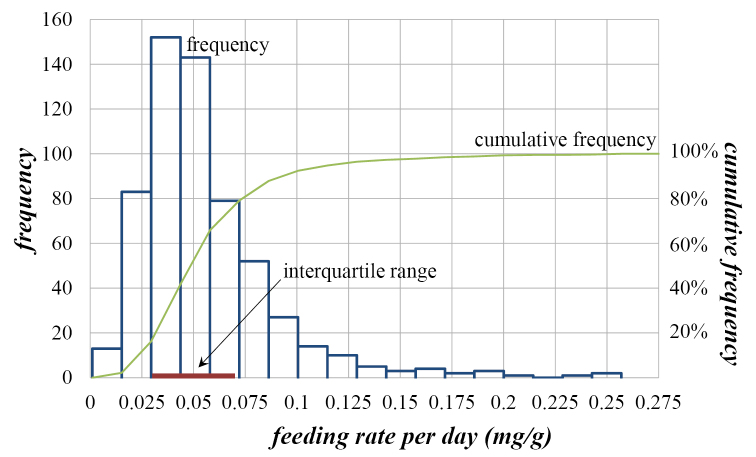
*Porcellio scaber*: Frequency distributions and interquartile range of feeding rate per day ([0.03< *FR*<0.07] mg/g).

**Figure 3.**
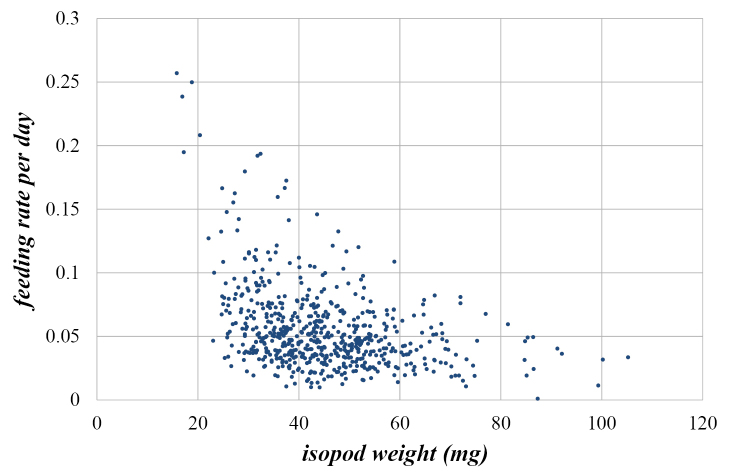
Scatter graph of *Porcellio scaber* weight and feeding rate per day (*r*=-0.38; *p*<0.001).

Variation of feeding rate per day (*FR*), judging by the coefficients of variation, is twice the variation of the isopod weight (*IW*) at the beginning of the experiment (Table 1). Table 1 and especially Figs 1 and 2 reveal that the observed variables of the populations are not normally distributed (Kolmogornov-Smirnov test: for both variables *p*<0.001). Figs 1 and 2 show a frequency distribution histogram, polygon of cumulative frequency, as well as the interquartile range (*Q*_3_-*Q*_1_) of animal weight and their feeding rate. Considering the weight of animals, there were less higher-weight animals than the mean weight (Fig. 1). Similarly, there were less animals with higher feeding rate per day then the mean feeding rate per day (Fig. 2). However, the results of the correlation analysis showed that *Porcellio scaber* weight and feeding rate per day were negatively correlated (Fig. 3) indicating that higher-weight animals consumed less food per day. The Pearson coefficient of correlation was significant at *p*<0.001 (T=-10.03).

Table 2 shows confidence intervals for the first (*Q*_1_) and the third (*Q*_3_) quartile for analysed variables and for different probability Pr (Pr=1-α) assuming that *Q*_1_and *Q*_3_ in the samples are normally distributed.

**Table 1.** Descriptive statistics for animals (*Porcellio scaber*) from laboratory-feeding experiments.

Statistics	Isopod weight at the beginning of experiment (in mg; *IW*)	Feeding rate per day (in mg/g; *FR*)
Number (*n*)	594	594
Minimum (*min*)	15.800	0.001
Maximum (*max*)	105.200	0.257
Mean (*m*)	44.800	0.055
Variance (*s*^2^)	177.672	0.001
Standard deviation (*s*)	13.329	0.033
Coefficient of variation (*CV*)	0.298	0.599
Skewness (*γ*_1_)	1.033	2.251
Kurtosis (*γ*_2_)	1.879	7.916
First quartile (*Q*_1_)	35.400	0.035
Median (*Me*)	43.050	0.048
Third quartile (*Q*_3_)	52.125	0.068

**Table 2.** *Porcellio scaber*: Confidence intervals for *Q*_1_ and *Q*_3_.

Isopod weight at the beginning of experiment (in mg; *IW*)	Feeding rate per day (in mg/g; *FR*)
Pr[34.28 < *Q*_1_ < 36.40]=0.95	Pr[0.033 < *Q*_1_ < 0.037]=0.95
Pr[50.93 < *Q*_3_ < 53.14]=0.95	Pr[0.065 < *Q*_3_ < 0.071]=0.95
Pr[33.78 < *Q*_1_ < 36.80]=0.99	Pr[0.032 < *Q*_1_ < 0.037]=0.99
Pr[50.43 < *Q*_3_ < 54.00]=0.99	Pr[0.062 < *Q*_3_ < 0.073]=0.99
Pr[33.48 < *Q*_1_ < 37.01]=0.999	Pr[0.032 < *Q*_1_ < 0.038]=0.999
Pr[49.83 < *Q*_3_ < 54.20]=0.999	Pr[0.062 < *Q*_3_ < 0.074]=0.999

In the case where the probability Pr=0.99 was chosen, feeding rate per day (*FR*) of the central 50% of the control population was situated in the interval [0.032,0.073] mg/g; Pr[0.032< *FR*<0.073]=0.99. In addition, if probability was lower (Pr=0.95), the result for the interquartile range of feeding rate per day was similar (Pr[0.033< *FR*<0.071]=0.95). Therefore, the central 50% of values of feeding rate per day were in the interval [0.03< *FR*<0.07] mg/g.

The reference values for our laboratory control *Porcellio scaber* was between 0.03 and 0.07 mg/g of feeding rate per day ([0.03< *FR*<0.07] mg/g) when their weight at the beginning of the test was in the range between 34 and 54 mg ([34< *IW*<54] mg) (Figs 1–2).

## Discussion

Terrestrial isopods of the species *Porcellio scaber* are among the most frequently used species in (eco)toxicity testing, and feeding parameters are among more relevant ecotoxicity endpoints (Drobne 1997). They indicate organism level responses and potential ecological implications. A significant obstacle of using feeding parameters as an endpoint is their high variability already in a control population (Drobne and Hopkin 1994). To overcome this shortage, we propose reference benchmark values for feeding rate, which we define as a characteristic values for control animals. According to our results, the feeding rate of *Porcellio scaber* per day of the central 50% of the control population was between 0.03 and 0.07 mg food/g animal/day. The deviation from these values might indicate an altered physiological state of animals.

Benchmark values for measured parameters in control animals allow direct comparison of measurement results in proficiency testing (round robin testing) performed by different laboratories (ASTM E1301-95). In addition, benchmark values for control animals could also be used in hazard and risk assessments to define data quality (Hobbs et al. 2005).

The data for feeding rate were not-normally distributed and nonparametric statistics must be used for further analysis. Not-normal data distribution can be explained by the fact that some animals could eat less or even stop eating, but tested animals could not eat above a certain limit. The coefficient of variation allowed us to compare the variation of different data. When we correlated feeding and isopod weight, it appeared that the correlation is relatively low suggesting that feeding behaviour of animals depends not only on weight but also on other parameters, which have not been tested yet or are even unknown. Our results suggest that data for feeding rate have higher variability as data for isopod weight.

Information on both technical and biological variance is of major importance in interpretation of the results of toxicity testing and in assessing their quality. Technical variance is attributed to performance of experiments and experimental conditions, while the biological source of variance is an inherited biological variability of the measured parameter. In our tests, most of the variance originated from the biological variability, and the technical variance was presumed to be low due to controlled and constant experimental conditions. Our data confirm that variance is an intrinsic characteristic of feeding parameters. Analysing and understanding the variance is of significant importance in the decision-making process used to set proper safety factors (Warren-Hicks et al. 2000).

In a single species laboratory test with *Porcellio scaber* several endpoints are commonly assessed. These are biochemical biomarkers, histopathological changes, behavioural responses and physiological alterations as well as different organism level responses including moulting, mortality and growth. The selected biomarkers vary in sensitivity, ease of observation, reproducibility, repeatability and ecological importance (Jemec et al. 2010). Conventional measures of toxicity such as growth, reproduction and life-cycle have high ecological relevance, but are difficult to assess under controlled laboratory conditions. Biochemical endpoints are very sensitive and respond quickly but they have limited ecological relevance (Jemec et al. 2010). Feeding parameters are intermediate between these endpoints. They are less sensitive as biochemical biomarkers, but still are readily reproducible and repeatable under controlled laboratory conditions (Drobne and Hopkin 1994). Literature data provide evidence on reduced feeding parameters after exposure of *Porcellio scaber* to metals, biocides or veterinary drugs in a dose-dependent manner (for summary see: Novak et al. (2012, 2013). However, in case of the pesticide diazinon, an increased but dose-independent feeding rate was reported (Stanek et al. 2006). After exposure of *Porcellio scaber* to silver nanoparticles in our laboratory, the feeding rate increased, but again not in a dose-dependent manner. These data indicate that feeding behaviour dynamics in isopods can be changed by different food additives. We suggest that in subchronic exposures, the benchmark values for feeding parameters as well as the coefficients of data variability may support the elaboration of lowest observed adverse effect concentrations, and data interpretation should not be based solely on statistical tests of significance. In addition, this approach could also improve significantly the quality of data.

Feeding rate of terrestrial isopod *Porcellio scaber* either decreases or increases due to food additives. The significance of this change is difficult to assess when a parameter has high background variability. We suggest using reference, benchmark data for feeding parameters and coefficients of variation for feeding rate to: (a) discriminate between animals with altered feeding parameters with respect to a control in addition to existing statistical tests; (b) assess the data quality in future experiments by comparing of the coefficient of variation and the established range for feeding parameters in control animals. In addition, establishing also reference, resting values for other parameters analysed in toxicity tests could increase the relevance of toxicity data such as ECx, LOEC, NOEC, and could assist significantly in quality control of the data.
